# The aftermath of the interplay between the endoplasmic reticulum stress response and redox signaling

**DOI:** 10.1038/s12276-021-00560-8

**Published:** 2021-02-08

**Authors:** Kashi Raj Bhattarai, Thoufiqul Alam Riaz, Hyung-Ryong Kim, Han-Jung Chae

**Affiliations:** 1 School of Pharmacy and Institute of New Drug Development, Jeonbuk National University, Jeonju, 54896 Republic of Korea; 2 Department of Pharmacology and Institute of New Drug Development, Jeonbuk National University Medical School, Jeonju, 54896 Republic of Korea; 3 College of Dentistry, Dankook University, Cheonan, 31116 Republic of Korea

**Keywords:** Mechanisms of disease, Cell biology

## Abstract

The endoplasmic reticulum (ER) is an essential organelle of eukaryotic cells. Its main functions include protein synthesis, proper protein folding, protein modification, and the transportation of synthesized proteins. Any perturbations in ER function, such as increased demand for protein folding or the accumulation of unfolded or misfolded proteins in the ER lumen, lead to a stress response called the unfolded protein response (UPR). The primary aim of the UPR is to restore cellular homeostasis; however, it triggers apoptotic signaling during prolonged stress. The core mechanisms of the ER stress response, the failure to respond to cellular stress, and the final fate of the cell are not yet clear. Here, we discuss cellular fate during ER stress, cross talk between the ER and mitochondria and its significance, and conditions that can trigger ER stress response failure. We also describe how the redox environment affects the ER stress response, and vice versa, and the aftermath of the ER stress response, integrating a discussion on redox imbalance-induced ER stress response failure progressing to cell death and dynamic pathophysiological changes.

## Cell biology: Protein misfolding induces vicious cycle of cellular stress

The endoplasmic reticulum (ER), a cellular organelle responsible for protein folding, is sensitive to chemical imbalances that can induce stress, leading to cell death and disease. Researchers in South Korea, led by Han-Jung Chae from Jeonbuk National University in Jeonju and Hyung-Ryong Kim from Dankook University in Cheonan, review how the ER counters changes in its environment that spur protein folding defects by activating a series of signaling pathways, known collectively as the unfolded protein response. Redox imbalance, may fail adaptive ER stress response that can damage the ER and surrounding mitochondria by modifying cysteine residues. The interaction between the two stress systems, ER stress and oxidative stress, has profound negative impacts on normal physiology. Targeting one or both of these stress mechanisms may therefore be an effective means of treating disease.

## Introduction

The endoplasmic reticulum (ER) functions as a tool for synthesizing and folding 30% of the proteome, but its usual function is easily affected by external factors^1–3^. The ER protein quality control system maintains cell homeostasis. The quality control system does not function with at least one-third of the polypeptides transported into the ER. The misfolded or unfolded proteins in the ER are retro-translocated to the cytosol for proteasomal degradation, which involves the ER-associated degradation (ERAD) pathway, whose primary function is protein clearance^4,5^. When ubiquitination and proteasomal degradation are impaired, the high levels of accumulated misfolded/unfolded proteins in the ER block the ER lumen^3,6^. In stressful environments, the demand for secreted and membrane proteins increases quickly, leading to increased protein synthesis levels that exceed the cellular protein degradation capability, resulting in protein accumulation^6^. Genetic, environmental, and nutrition-related insults induce imbalances in the ER quality control system or imbalanced ER proteostasis, leading to ER stress-dependent nonnative protein secretions. Dysregulation or disruption of the oxidation–reduction pattern of equilibration–disruption of redox functions, changes in calcium levels, or posttranslation can exacerbate the imbalance to a critical point, initiating ER stress-induced cell death^1,6,7^. In addition, defective autophagy, energy deficiency, and inflammatory stimulation contribute to the accumulation of misfolded proteins^6^. Because of the high protein load on the organelle or impaired ER quality control mechanisms, protein degradation may be disrupted, leading to high protein accumulation in the ER, inducing the unfolded protein response (UPR), and thus initiating a stress response^1,2^. Here, we explain canonical ER stress, noncanonical ER stress, and ER stress response failure to enhance our understanding of ER stress signaling. This review also discusses how ER stress signaling affects mitochondria and vice versa and cell fate determination.

## Endoplasmic reticulum stress and the unfolded protein response

**Fig. 1:**
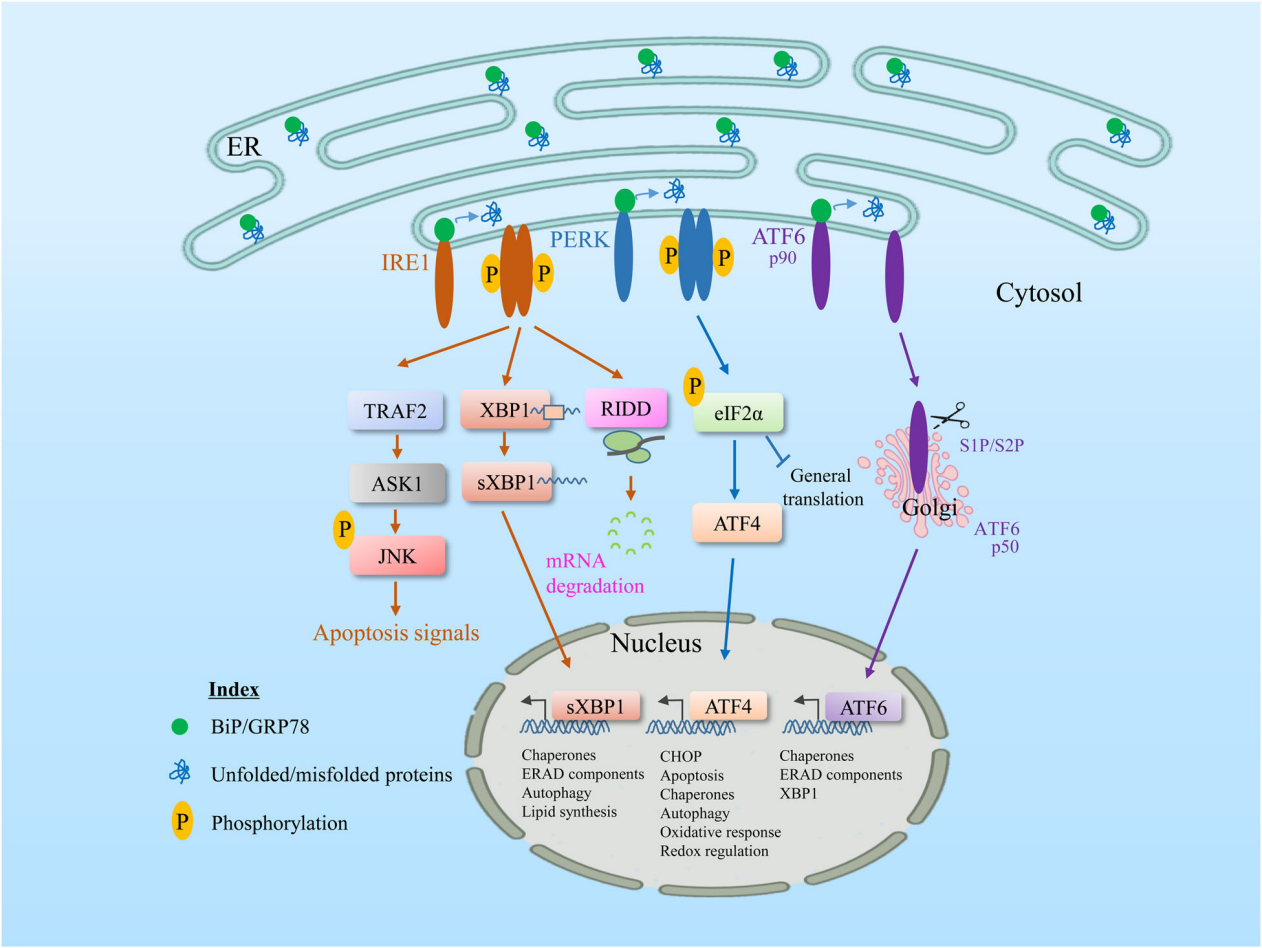
General unfolded protein response pathway during ER stress. GRP78/BiP, an ER chaperone, is closely associated with three sensors of the UPR, IRE1, PERK, and ATF6, inhibiting them under normal physiological conditions. Upon ER stress or misfolded protein accumulation, GRP78 dissociates from all these UPR transducers and permits stress sensors to activate downstream signaling. A different signal transduction system activates each pathway. The most conserved signal transducer, IRE1 (which contains a serine/threonine kinase and an RNase domain on its cytosolic side), undergoes homodimerization and autophosphorylation, and its activation mediates the activation of its RNase domain to produce spliced XBP1 mRNA, which is the active isoform of XBP1 that is translocated to the nucleus to increase UPR target genes, including chaperones and ERAD. The RNase domain of IRE1 also regulates the RIDD (regulated IRE1-dependent decay) pathway, where IRE1 degrades ER membrane-localized mRNAs through its RNase activity, resulting in a reduction in the amount of protein imported into the ER lumen. Similarly, during ER stress, the cytosolic domain of IRE1 interacts with TRAF2 and activates the downstream kinase ASK1, enhancing the activated JNK pathway and triggering apoptosis. Similarly, activation of PERK increases the phosphorylation of the alpha subunit of the translation protein eIF2, which attenuates protein translation to reduce ER protein overload, while paradoxically upregulating ATF4 mRNA, which targets the activation of proapoptotic CHOP and other UPR target genes. Upon sensing ER stress, a third UPR transducer, ATF6 alpha, is translocated to the Golgi apparatus, where it undergoes cleavage by site-1 and site-2 proteases. The cleaved fragments are then translocated to the nucleus and activate the transcriptional target genes of ATF6, including chaperones and XBP1. Adaptive response failure may not resolve ER stress and may upregulate UPR signaling to induce apoptosis.

When the ER becomes overloaded, cells experience ER stress disorder, characterized by the accumulation of misfolded proteins within the ER lumen. Various conditions, such as nutrient deprivation, hypoxia, viral infection, exposure to oxidative stress, and calcium depletion, can affect the homeostasis of cellular compartments and cause ER stress. Cells developed an evolutionarily conserved signal transduction mechanism called the UPR to resolve imbalanced ER protein folding, with the primary purpose of restoring ER homeostasis. In summary, the UPR is a signaling mechanism that is activated in cells in response to ER stress^8^. In general, UPR signaling is controlled by three main ER transmembrane-associated sensor proteins, namely, inositol-requiring enzyme 1 alpha (IRE1α), protein kinase R-like endoplasmic reticulum kinase (PERK), and activating transcription factor 6 (ATF6)^6^. The ER-resident chaperone BiP/GRP78 strikes a complex balance between unfolded and intraluminal (to be folded) proteins and the three ER stress sensors. Accumulated unfolded proteins sense disrupted equilibrium, resulting in GRP78 dissociation from the ER stress sensors that cooperate extensively in protein folding. Here, we describe the major signal transduction pathway of the ER stress response (Fig. 1).

## ER stress signaling pathway

The three main UPR branches may act synergistically or differentially according to the activation mode with different strength and time courses^9^. Each branch activation causes b-ZIP transcription factors to function individually or jointly to activate the downstream target genes of the UPR. Activated UPR controls nonspecific transcriptional ER processes, such as mitochondrial function, amino acid metabolism, cellular redox status, and small molecule transport. The mechanisms of the UPR involving transcriptional activation ensure a certain degree of ER stress (acute or prolonged), which can alter several contributing mechanisms and ideally allow enhanced efficiency of the secretory pathway to alleviate the stressful stimulus. If the adaptive response cannot restore protein folding homeostasis, the UPR signals are continuously emitted and eventually are converted to alternative signals called “terminal UPR” signals, which promote apoptotic signaling^10^.

The most conserved ER stress signaling branch is IRE1α, and its activation mechanism has been extensively investigated. IRE1α is a type 1 transmembrane bifunctional protein kinase with three domain areas: the luminary N-terminal domain, endoribonuclease cytosolic (RNase) domain, and serine/threonine kinase cytosolic domain^11^. Responding to the accumulation of unfolded proteins during ER stress conditions, IRE1α dimerizes and trans-autophosphorylates, leading to the activation of the cytosolic region RNase domain, which is an activating result of a conformational modification caused by the excision of a 26-nucleotide intron from mRNA that encodes the transcription factor XBP1 (ref. ^12^). The consequence of this splicing event is a frameshift in the mRNA, and the transcription factor XBP1 thus becomes active and stable. Subsequently, XBP1 is translocated to the nucleus where it upregulates prosurvival target genes^13^ to generate multiple cell survival factors. XBP1 also increases the protein secretion rate in ER and Golgi compartments. Furthermore, IRE1α activation triggers the degradation of other ER-localized mRNAs in a process called regulated IRE1-dependent decay (RIDD), in addition to producing stable spliced transcription factors, such as XBP1s^14^. However, IRE1 interacts with TRAF2 to activate the inflammatory response and cellular apoptosis-associated protein kinases, especially apoptosis signal-regulating kinase 1 (ASK1), which leads to JNK activation^15^. In addition, IRE1–TRAF2 complexes recruit IκB kinase, which results in the degradation and phosphorylation of IκB, and consequently the translocation of nuclear factor-κB (NF-κB) to the nucleus to control inflammatory gene transcription^16^.

PERK, an ER-resident transmembrane kinase, is the second UPR signaling branch. PERK phosphorylates the downstream substrate of eukaryotic initiator of translation factor 2α (eIF2α) at serine 51 under ER stress conditions and contributes to the inhibition of protein synthesis in the ER lumen. When initiating translation, eIF2 constitutes a Met–tRNAi–GTP ternary complex that binds the 40 S subunit of 43 S PIC^17^. During eIF2α phosphorylation at Ser51, the binding affinity of eIF2 to its guanine nucleotide exchange factor, called eIF2B, which blocks the conversion of eIF2-GDP to eIF2-GTP, and thus prevents translation, is significantly enhanced^18,19^. PERK thus helps to reduce protein streaming into the ER, alleviating ER stress. However, when eIF2α is limited, mRNAs with short open reading frames at 5′ untranslated regions are favored. The transcription factor ATF4 is the produce of one of the encoded genes that induces translation. CHOP (transcription factor C/EBP homologous protein) and GADD34 (growth arrest and DNA damage-inducible 34) are two major target genes induced by ATF4. CHOP is a transcription factor that regulates the genes encoding apoptosis components. Thus, at modest signaling levels, the PERK branch is highly protective, but it can also activate cell death pathways. This dualism is likely to occur at the phosphorylated eIF2α stage and is demonstrated by the results of its unique phosphatase modulation. GADD34 encodes the phosphatase PP1c PERK-inducible regulatory subunit that counteracts PERK by dephosphorylating eIF2α. The GADD34–PP1c complex defends cells against ER stress by expanding low-level phosphorylation of eIF2α and is selectively inhibited by either small molecules or GADD34 deletion.

The third ER stress sensor, ATF6, is a transmembrane transcription factor with an N-terminal cytosolic domain and a C-terminus in the ER lumen that is regulated by ER stress. After sensing ER stress, ATF6 can activate the transcription of ER molecular chaperones. Under ER stress conditions, the S1P and S2P endopeptidases transfer ATF6α for cleavage in the Golgi system, thereby releasing the activated ATF6α form^20^. The cleavage of ATF6 at a juxta-membrane site contributes to its discharge into the cytosol^21^. The 50-kDa soluble ATF6 cytosolic fragment is then transported into the nucleus, where it associates with ATF/cAMP response elements and endoplasmic reticulum stress response elements (ERSE-I)^22^. Consequently, UPR target genes, including XBP1, CHOP, and GRP78, are regulated and activated transcriptionally^23^.

In addition to these abovementioned canonical ER stress pathways, other pathways or specific elements of the UPR have been linked as determinant of cell fate acting independent of the classical UPR, a process known as noncanonical ER stress. Mainly, the integrated stress response (ISR), translocation of proteins into the ER, extracellular-signal-regulated kinase reactivation, ERAD, ERphagy, and other pathways have been linked to the noncanonical ER stress response^24^. The ISR is an evolutionarily conserved program affecting homeostasis that is activated by different pathological conditions, such as hypoxia, viral infection, amino acid deprivation, and glucose deprivation, or cell-intrinsic factors, including ER stress caused by the accumulation of unfolded/misfolded proteins in the ER^25^. The ISR-inducing stressors activate PERK, PKR (double-stranded RNA-activated protein kinase), GCN2 (general control nonderepressible 2), and HRI (heme-regulated inhibitor) kinases, which are linked to the phosphorylation of eIF2α at Ser51 to inhibit the translation of new proteins. Simultaneously, it initiates the translation of specific mRNAs of ATF4, the main effector of ISR. Thus, ATF4 is a critical transcription factor inducing the expression of genes involved in autophagy, antioxidant response, amino acid metabolism, and cell death^26^. Recently, the PERK pathway was identified as an attenuator of IRE1 signaling via protein phosphatase RNA polymerase II-associated protein 2, which suppresses IRE1 oligomerization and RNase activity, inhibiting the production of spliced XBP1 and ERAD^27^. Other pathways that are linked with noncanonical ER stress have been described in previously published articles^24,25^. In addition to canonical or noncanonical ER stress responses, accumulating evidence suggests that ER stress response failure contributes to the development of pathogenesis, which we discuss in a separate section.

## ER stress leads to disease progression

During ER stress, a decrease in the capacity of cells to recover misfolded or unfolded proteins may induce cellular dysfunction and disease. The diminished capacity of cells to fold secreted or membrane proteins, the decreased ability to identify or respond to misfolded proteins, or the increased load of misfolded proteins in the ER leads to ER stress and causes several diseases. Improper activation of the UPR can be dangerous because it can destroy the cell or protecting the cell against death (e.g., during neoplastic transformation or viral infection). Each of these conditions has been shown to trigger cellular or organ damage in humans or model organisms under pathological conditions. Table 1 addresses the ER stress-related proteins and their associated pathways that lead to the development of different diseases.

**Table 1:** ER stress and associated diseases.

Diseases and conditions	Proteins involved	Mechanism	References
Alzheimer’s disease (AD)	GRP78, CHOP/GADD153, PERK, eIF2α, and IRE1α	ER stress proteins such as GRP78 and phosphorylated forms of PERK, eIF2α, and IRE1α in AD are studied. During prolonged ER stress in AD brains, proapoptotic components such as ATF4-CHOP are highly increased. Evidence suggests that the expression of not only GRP78 but also of PDI, target genes of XBP1, is increased in AD. XBP1 is increased in AD and caspase-3, 4, and 12 are also increased in AD. However, the UPR apoptotic pathway was not activated in a transgenic aged mouse model of AD (Tg2576 mice), suggesting that defective UPR activation is involved in AD pathogenesis.	^119,120^
Parkinson’s disease	Parkin	A Parkin substrate is deposited in the ER to induce ER stress.	^121^
Amyotrophic lateral sclerosis	SOD1	ER stress is induced by the aggregation of SOD1 mutants.	^122^
Bipolar disorder	GRP78, eIF2α, and CHOP	Dysfunction or impairment of the ER stress response is associated with decreased cellular resilience in bipolar disorder; however, the precise mechanisms of this study are lacking.	^123,124^
Nephrotoxicity	CHOP, caspase-12, PERK, and GRP78	ER stress-mediated apoptosis and the inhibition of autophagy lead to nephrotoxicity. In addition, the activation of CHOP and cleavage of caspase-12 induce an ER stress response in drug-induced renal injury (e.g., paracetamol).	^125–127^
Type 1 diabetes	IRE1α, JNK-AP1, IL-1β, caspase-1, caspase-2, CHOP, DR5, caspase-12, and TXNIP	IRE1α-associated β cells cause damage by activating the apoptotic pathways. The JNK-AP1 and NFkB pathways exacerbate insulitis by inducing the infiltration of immune cells and activating proinflammatory genes. RIDD-mediated insulitis and β-cell death is induced by the activation of IL-1β, caspase-1, and caspase-2; β-cell death is also induced through IRE1α/JNK/CHOP/DR5 and caspase-12 activation.	^128–130^
Type 2 diabetes	JNK, IRS-1, and XBP1	Obesity-induced ER stress leads to the hyperactivation of c-Jun N-terminal kinase (JNK) and subsequent serine phosphorylation of insulin receptor substrate-1 (IRS-1), which promotes insulin resistance.	^89,131^
	CREB-regulated transcription coactivator 2 (CRTC2) and ATF6	Acute increases in ER stress cause CRTC2 dephosphorylation and nuclear entry, which enhances the expression of ER quality control genes via ATF6α, and therefore, ATF6 impairs gluconeogenesis.	^132^
	CHOP	Hyperglycemia and free fatty acids induce β-cell death via CHOP.	^133^
Diabetic cardiomyopathy	GRP78, GRP94, IRE1, ATF6, and PERK	ER stress induction by hyperglycemia, hyperlipidemia, homocysteine, or ischemia may cause cardiac inflammation/remodeling or cardiac dysfunction and cardiomyopathy.	^134^
Atherosclerosis	CHOP	Relevant stimuli of atherosclerosis induce macrophage death via CHOP.	^135,136^
	CHOP	Endothelial and smooth cell death through CHOP is caused by oxidization of phospholipids, high cholesterol levels, and hyperhomocysteinemia.	^137^
Liver diseases	CHOP, ATF6, IRE1, GRP78, and SREBP	Alcoholic and nonalcoholic liver diseases are known to be induced by ER stress. ER stress promotes the activation of SREBP-1c and thus promotes lipogenesis. Alcohol-induced ER stress activates CHOP-mediated apoptosis of hepatocytes. ER stress is also involved in hepatocellular carcinoma where ATF6 and IRE1 pathways, including GRP78, are involved.	^123,138^
Rheumatoid arthritis (RA)	IRE1α, IL-β, IL-6, and TNFα	RA boosts proinflammatory cytokines such as IL-β, IL-6, and TNFα in both infiltrated macrophages and fibroblast-like synoviocytes. IRE1α increases inflammation and angiogenesis through the mediated activation of infiltrated macrophages via toll-like receptors, and enhances synovial fibroblasts survival by upregulating ER degradation genes.	^139–142^
Systemic lupus erythematosus	IRE1α, JNK, XBP1s BCL-2-associated X protein, and CHOP	IRE1α/JNK/BCL-2-associated X protein and IRE1α/XBP1s/CHOP pathways lead to apoptosis in specific tissues.	^143,144^
Vitiligo	IRE1α, XBP1s, and TNFα	Cytokine production through IRE1α/XBP1s causes melanocyte loss. Melanocyte stem cell differentiation is inhibited by the IRE1α/XBP1s/TNFα pathway.	^145,146^
Inflammatory bowel disease	IRE1α, JNK, and NFκB	JNK- and NFκB-mediated cytokine production induces IRE1α to induce the secondary consequences of this disease.	^147,148^
Systemic sclerosis (scleroderma)	IRE1α, XBP1s, GRP78, JNK, AP1, and NFkB	The activated IRE1α/-XBP1s pathway leads to ER biogenesis, which facilitates the adaptation to an increased demand for myofibroblast protein folding. In the IRE1α/XBP1 pathway, ER chaperones such as GRP78 may contribute to efficient protein folding. The pathway degrades IRE1α/RIDD miRNA-150, a repressor of α-SMA and collagens I and IV expression, resulting in enhanced IRE1α/JNK/AP1 fibrosis, and IRE1α/NFkB pathways may involve systemic sclerosis and the expression for endothelin-1.	^149,150^
Viral infection	PERK, ATF6, and IRE1	These three pathways are all involved in hepatitis C infection and HIV progression.	^151^
Hepatitis B virus (HBV) infection	GRP78 and PERK	Hepatitis B surface antigen stimulates the UPR through the PERK pathway and induces GRP78 expression.	^152^
Hepatitis C virus (HCV) infection	IRE1 and XBP1	HCV suppresses the IRE1/XBP1 pathway to increase the synthesis of viral proteins and increase the survival of the virus in infected hepatocytes.	^153^
Alcoholic liver disease	GRP78, GRP94, and SREBPs	High intracellular homocysteine levels increase the expression of various UPR genes, including GRP78, GRP94, HERP, and RTP. ER stress triggers lipid biosynthesis dysregulation by activating SREBPs that lead to increased hepatic biosynthesis and cholesterol and triglyceride production.	^154^
Ischemia	ATF6, IRE1, PERK, and CHOP	Brain ischemia contributes to ER stress in neurons and triggers the ATF6, IRE1, and PERK pathways, leading to neuron apoptosis mediated by CHOP.	^155,156^
Tumorigenesis and cancers	GRP78, XBP1, CHOP, and IRE1	GRP78 and XBP1 are involved in protective and proliferative effects in the tumor cells. The loss of CHOP production increases tumor survival in colon cancer. IRE1 mutations are involved in breast and lung malignancies. The downregulation of UPR genes is observed in prostate cancer.	^151,157,158^
Aging-associated diseases	UPR-related proteins	Impaired UPR, decreased cell survival, and increased apoptosis rate.	^88,115,159^

## UPR regulation from the redox perspective

ROS are normally small, short-lived, and highly reactive molecules^28,29^. They can be free radicals derived from oxygen, including anionic superoxide (O_2_•−) or the hydroxyl radical (OH•) or nonradical molecules, e.g., hydrogen peroxide (H_2_O_2_). ROS are generated in cells with a variety of antioxidant defenders in equilibrium. In this case, enzyme scavengers such as superoxide dismutase (SOD), glutathione peroxidase, peroxiredoxins, catalase, and nonenzyme scavengers, such as vitamin C and E, glutathione (reduced glutathione (GSH)), lipoic acid, carotenoids, and iron chelators are involved^28^. ROS are involved in regulating normal physiological functions by activating various cellular signaling pathways and transcription factors, including phosphoinositide 3 kinase (PI3K)/Akt, mitogen-activated protein kinase, nuclear factor erythroid 2-related factor 2 (NRF2)/Kelch-like ECH-associated protein 1, NF-κB, and tumor suppressor p53, supporting cellular survival or death processes^30,31^. The transduction of redox-controlled signals is often carried out through reversible thiol protein oxidation. However, further research is needed to understand the physiological relevance, and redox signaling mechanisms at the cellular level to determine the ROS threshold and level and the severity of oxidative stress^29,31^.

## ER and mitochondrial ROS regulate redox signaling mechanisms

Protein is commonly required to implement biological activity in a particular three-dimensional structure^32,33^. The indigenous conformation of several proteins, particularly secretory and membrane proteins, is stabilized by intramolecular disulfide bonds^34^. The ER is a reticular organelle that folds and changes the action of nascent proteins translocated by cytoplasmic ribosomes^35^. In the ER, H_2_O_2_ is produced as a byproduct in the protein folding process, and hence, the ER retains relatively high ROS levels^36,37^. The ER redox state is closely related to ER homeostasis, and proper functioning of the ER is due to disulfide bridge formation during protein folding. The regulation of the redox state by the ER and mitochondrial pathways is described below.

## ER pathway

Intramolecular disulfide bonding, oxidative processes, and possibly the most common posttranslational modification are characteristics of oxidative protein folding (OPF)^38^. Disulfide bond formation is primarily catalyzed by PDI, which has four Trx domains (a, b, b′, and a′) and a KDEL ER retention series c-domain^39,40^. The redox state of the CGHC motifs in the a-domains in PDI determines whether the functions of oxidases or isomerases are triggered^41,42^. The noncatalytic b′ domain identifies unfolded and incorrectly folded proteins via exposed hydrophobic patches on a protruding part of the protein^43^. The oxidation of nascent proteins is carried out for catalytic purposes by reducing PDI via the CGHC active site^42^. The next reaction is the reoxidation of PDI active sites^44^. H_2_O_2_ is the principal source for oxidation, in addition to a byproduct produced by O_2_, which ER oxidoreductases use to oxidize PDI. In peroxidases, H_2_O_2_ may be used to shape disulfide bonds that can reoxidize PDI or maintain redox homeostasis in the ER.

Similarly, ERO1 is a highly conserved flavoprotein with two cysteine pairs situated on a flexible loop and on a CXXC motif that is proximal to the flavin adenine dinucleotide cofactor^45^. O_2_ is used as a sulfhydryl electron accepter by ERO1 to catalyze the PDI disulfide bond formation that produces H_2_O_2_. This process is essential to ensure PDI isomerase activity for oxidizing reduced PDI^36,37^. To avoid ROS overproduction, yeast ERO1p and mammalian ERO1α and ERO1β are closely regulated to maintain ER redox homeostasis or proper protein folding^39^.

**Fig. 2:**
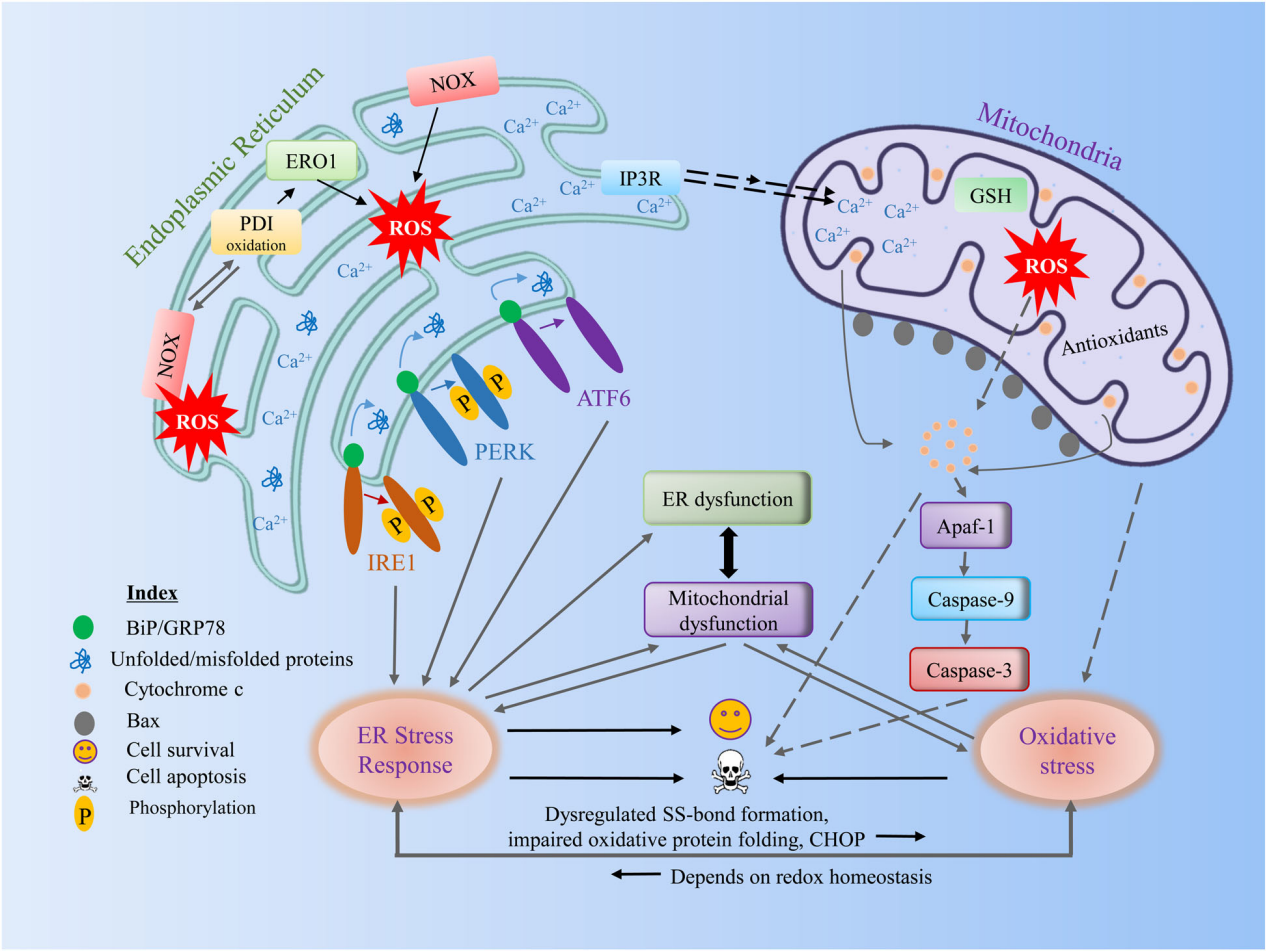
Cross talk between components of ER stress and the redox signaling pathway. During various pathological conditions, unfolded or misfolded proteins are increased in the ER. During oxidative protein folding in the ER, ROS are generated during electron transfer between PDI and ERO1α. ROS associated with UPR signaling can activate an antioxidant response, such as Nrf2 or can increase ROS generation by activating ERO1 or NOX. Furthermore, ROS are increased in the ER through the association of PDI with ROS, generating Nox4. Although the major site of calcium is in the ER, under stress conditions, calcium may flow to the mitochondrial outer membrane through calcium release channels, such as IP3R or RyR. Increased calcium overload in mitochondria subsequently increases ROS generation. The increased calcium load and ROS in mitochondria may lead to opening of the mitochondrial permeability transition pore, which may cause the release of proapoptotic factors. High oxidative stress during this condition is critical for inducing mitochondrial dysfunction and vice versa. Overall, we suggest that the ER stress response can induce ER or mitochondrial dysfunction, which may increase oxidative stress by dysregulating disulfide bond formation, impairing oxidative protein folding, or the inducing certain UPR genes (e.g., CHOP) where oxidative stress is reversible, which may depend on redox homeostasis (see the text for more details). ER endoplasmic reticulum, ROS reactive oxygen species, PDI protein disulfide isomerase, ERO1α endoplasmic reticulum oxidoreduction 1α, NOX NADPH oxidase, IP3R inositol 1,4,5-trisphosphate receptors, RyR ryanodine receptors.

Although ERO1 is important for oxidizing protein dithiols in yeast, ERO1α and ERO1β double knockout results in only a mild ERO1β phenotype that compromises mammalian oxidative folding of proinsulin^46,47^. In the absence of these flavoproteins, this surprising finding raises the question, What sustains oxidative folding? Similar to ERO1, quiescin sulfhydryl oxidase (QSOX), a flavoprotein containing an ERV/ALR domain fused with a domain, such as Trx, catalyzes the formation of disulfide by coupling disulfide oxidation with oxygen reduction to H_2_O_2_. It can also bypass a disulfide exchange reaction catalyzed by PDI due to its unique structure^48,49^. In vitro, QSOX can transport disulfides to the first thioredoxin domain between the ERV domain and then exchange the disulfide with substrate proteins^50^. Several NADPH oxidases (NOXs) are positioned on the ER membrane, where ROS generation is catalyzed^51,52^. For example, Nox4, which has been shown to be in the ER membrane, produces H_2_O_2_, which can cause apoptosis after extended ER stress^52^ (see Fig. 2).

## Mitochondrial pathway

Mitochondria are among the key sources of ROS production, where the process of oxidative phosphorylation produces unpaired electrons that interact with O_2_ and increase the production of highly reactive free radicals. While ROS are produced as byproducts of oxidative phosphorylation, a widely discussed long-standing issue has been the specific site(s) in the electron transmission chain critical for ROS generation. Under physiological conditions, complex I is usually considered the key site for the development of mitochondrial ROS, where O_2_•− on the side of the mitochondrial matrix is formed with rapid dismutation to H_2_O_2_ by SOD^53,54^.

Complex III is also classified as O_2_− producing machinery^15^^,^^16^. Under ischemic and apoptotic conditions, the development of O_2_•− in complex III is shown to be induced by the inhibition and reduction of the electron transport system^55^. Cellular ROS production is largely supported by NOX in certain cells, such as phagocytic neutrophils, nonphagocytic fibroblasts, smooth muscle, and vascular endothelial cells^56–58^. Recently, Nox4, a Nox isoform, was found to be expressed in the mitochondria of chronic myeloid leukemia cells that overexpress the nonapoptotic protein BCL-2 (CEM/BCL-2)^59^ in the renal cortex of rats^60^ and of cardiomyocytes, and in membrane fractions enriched with mitochondria. However, to date, Nox4 activity in mitochondria has not been evaluated explicitly, but cytoplasmic Nox4 can participate in promoting the modulation of PKCε, MitoKATP, and thioredoxin-2 activity, leading to the upregulation of mitochondrial ROS production by the electron transport chain, which is redox sensitive^61^. Other mitochondrial proteins, including pyruvate dehydrogenase, α-ketoglutarate dehydrogenase^62^, and glycerol-3-phosphate dehydrogenase, and fatty acid β-oxidation^56^ were identified as being produced by the electron transport chain and the NOX family.

In addition to ROS generation, mitochondrial-specific nitric oxide synthase (NOS) can lead to •NO development. The effect of nitric oxide (NO) production on mitochondrial functions is debated, particularly the amount of NO produced and the conditions in which it is generated. Mitochondrial respiration, even with moderate levels of •NO, can be partially inhibited, causing an increase in mitochondrial ROS output. The effect is depolarization of mitochondria, resulting in the reduced mitochondrial uptake of calcium^63^. The inhibitory effect of •NO is mainly caused by the inactivation of cytochrome c oxidase (COX; complex IV)^64^ and rate-limiting component of the electron transport chain. As a double-edged sword, •NO has been shown to play a role in mitochondrial biogenesis, in which it is stimulated by •NO generation through the upregulation of cGMP-dependent expression of PGC1α, which in turn increases the expression of mitochondrial transcription factor A and NRF1, and induces increased mitochondrial biosynthesis in adipocytes and liver cells^65^.

Furthermore, treatment with inorganic nitrates, nitrites, or S-nitroso-2-mercaptopropionyl glycine ensures the heart mounts a defense with different cellular proteins and is protected against injury by temporary S-nitrosylation (SNO)^66^. In contrast, excessive generation of •NO may lead to severe tissue damage upon certain pathological stimuli. Since a reaction of nitroxyl anion (NO−) and molecular oxygen is also possible, peroxynitrite can be formed through an alternative route. Peroxynitrite (ONOO−) spreads across mitochondrial membranes and can cause oxidative damage to critical components in mitochondria by oxygenation, nitration, and nitrosation. ONOO− has significant impacts on mitochondrial metabolism, calcium homeostasis, and mitochondrial permeability transition^67^. ONOO− also uncouples eNOS, thereby increasing ROS-producing enzymes where mitochondrial ROS levels are also increased^67,68^.

## Redox-induced cross talk between the ER and mitochondria

The main sources of ROS are mitochondria, but accumulating evidence suggests that the ER also plays a critical role in regulating redox reactions. Therefore, it is important to study the elusive redox interaction between these two ROS sources. Through the emerging role of redox in calcium homeostasis, the relationship between different cellular ROS sources is suitably illustrated. The ER is a large calcium reservoir. The sarco/endoplasmic reticulum calcium ATPase (SERCA) continuously pumps calcium into the ER from the cytoplasm. Calcium release from the ER occurs via the ryanodine receptor and the 1,4,5-triphosphate receptor (IP3R) under normal and pathological conditions. Oxidation of these calcium transporting agents (due to elevated ROS levels during cellular dysfunction or pathological condition) causes net calcium efflux from the ER into the cytoplasm^69^, particularly the oxidation of luminal cysteine by ROS.

The work of Booth and colleagues^70^ highlights the complex relationship between ROS, calcium, the ER, and mitochondria. These authors demonstrated that mitochondria uptake of the calcium released from the ER, stimulating the mitochondrial ETC and accumulation of mitochondrial peroxide. Redox reactions involving the ER and mitochondria were suggested to culminate in cell death by apoptosis to clear cells with excessive ROS. Using a yeast model, Leadsham and colleagues revealed a clear interplay between mitochondrial dysfunction and the production of peroxide by ER-localized NOX^71^. This study gives rise to possible conflicts regarding the sources of ROS and the importance of the ER. The authors found that a reduction in COX activity contributes to increased cellular ROS levels. These observations suggest that the mitochondrial ETC is critical for the increase in superoxide. However, this increase was traced to ER-localized NOX.

There is an unusual redox cycle capable of combining oxidative folding with ROS accumulation in the ER. ER protein folding ability is regulated by the activity of the UPR: cells show increased chaperone levels when ER folding ability is affected^72^. Paradoxically, when the UPR is generated in the ER, ROS levels seem to increase, but ER stress is expected to be minimized by the UPR^69,72^. The reestablishment of ER homeostasis appears to have an inadvertent result on ROS production at face value; the increase in ER ROS levels is a possible byproduct of higher ERO1-PDI levels and UPR targeting of NOX enzymes. However, increased ROS may not be the only products of UPR induction, and ROS may signal and modify the cellular stress response (see Fig. 2). For example, cysteine residues in UPR sensors can increase or diminish UPR signals emitted through ROS-mediated oxidation^69^. Cells that demonstrate prolonged UPR activation (failure to recover ER homeostasis) may evoke mitochondrial activity to activate ER calcium release and thus induce apoptosis.

## Cellular fate during ER stress and redox imbalance

OPF, which is characterized by the production of intermolecular or intramolecular disulfide bonds, is the leading source of H_2_O_2_ in the ER^32,33,73^. In conjunction with PDIs, which play significant roles in OPF, ERO1 proteins, for instance, respond to how the ER can generate oxidative power^36,37^. To preserve redox homeostasis, H_2_O_2_ is used as a common oxidant in some oxidation disorders, such as peroxiredoxin 4, glutathione peroxidase 7/8, and ascorbate peroxidase, to ensure OPF^74,75^. Furthermore, GSH, the most common reducing agent in cells, contributes to the elimination of excessive ROS^76^. ROS homeostasis is important in the ER. Although OPF friendly, over accumulated ROS (referred to as oxidative stress) may disrupt the redox homeostasis of the ER, leading to the accumulation of malfunctioning proteins and causing ER stress^73^. Upon disruption of ER homeostasis (protein folding homeostasis or redox homeostasis), the UPR is stimulated to restore stress^77^. To restore protein folding ability, UPR sensors, including IRE1α, PERK, and ATF6α, are activated to induce complex pathway networks, including prosurvival autophagy mechanisms, antioxidant reactions, ERAD, and ER biogenesis^78,79^ and apoptosis and ferroptosis prodeath mechanisms^35^. Growing evidence suggests that ROS and redox signaling are profoundly involved in deciding cellular fate.

The most critical link between ER stress and redox regulation is composed of many sources of ROS (from the ER or mitochondria). These sources can interfere with ER protein folding and cause ER stress, which may stimulate the UPR to induce apoptosis^69^. Misfolded proteins in the ER, important sources of ROS, may lead to oxidative stress, given the role played by the formation of disulfide bonds in the ER. During ER stress, dysregulated disulfide bond formation or breakage may induce ROS generation and cause oxidative stress by depleting ER GSH, which may eventually lead to apoptosis^80^. Some UPR elements, such as CHOP, are also critical for inducing oxidative stress. The overactivation of ERO1α by CHOP during ER stress increases the ROS production. ERO1α also induces ER IP3R-mediated Ca^2+^ leakage, activating Ca^2+^ sensor kinase and Ca^2+^/calmodulin-dependent protein kinase II (CaMKII) in the cytosol, which results in the activation of proapoptotic pathways, including the mitochondrial membrane permeability transition and Fas^81–83^. ERO2α also causes Ca^2+^/CaMKII sensing kinase activation in the cytosol. CaMKII also induces NOX subunit Nox2, a positive feed-forward loop during ER stress^84^, and thus triggers the ROS oxidative stress. Since the process of protein folding depends on redox homeostasis, increased oxidative stress may compromise protein folding machinery, leading to the production of misfolded proteins and exaggerating ER stress^85^ (see Fig. 2). Overall, redox signaling mediators have important roles in generating ROS during ER stress, and mitochondria greatly contribute to the ROS generation^86,87^. Further research is required to study the in-depth mechanism by which ER and mitochondrial associations play roles in regulating redox reactions, which will help with treating diseases caused by protein misfolding.

## ER stress response failure

In addition to the ER stress response, ER stress response failure is of considerable interest to researchers in this field. ER stress coping mechanisms in cellular health have been established, but cell fate after ER stress response failure remains unclear. Our recent paper suggests that the ER stress response and ER stress response failure play divergent roles in cells^88^. Upon ER stress, the UPR, primarily functions to achieve cellular adaptation by maintaining ER homeostasis. However, the UPR and ER stress response may fail to alleviate cellular stress under severe stress or pathological conditions. During this condition, ER stress sensors may not be fully activated or aberrantly expressed, further downregulating the downstream signaling. This downregulation may diminish protein quality control and drive cells toward demise. We previously described that aging and its associated diseases, such as obesity and diabetes, may induce ER stress response failure, most likely under severe ER stress conditions. We speculate that various degrees of cellular stress can determine the degree of adaptive UPR, ER stress, and ER stress response or response failure, leading to either cell survival or cell death.

## How does ER stress response failure contribute to disease progression?

Although ER stress response failure has not been studied extensively, our recent paper suggests that this mechanism is associated with several diseases and is more specific to metabolic diseases, including obesity and diabetes^88^. Aging and its related metabolic disturbances can also induce ER stress response failure, where various complex mechanisms are involved. Several previous reports have revealed that ER stress is highly induced in an obesity context, such as in high-fat diet (HFD)-fed rodents or palmitate-exposed cells^23,89^. However, some contradictory data from studies of muscle show that while body fat and glucose intolerance are increased during HFD treatment, the UPR is not activated. The major UPR sensors, such as IRE1 and PERK, or other UPR elements, such as GRP78, calnexin, CHOP, ATF4, or XBP1s mRNA, are not changed after HFD exposure^90^. These data suggest that ER stress response failure can contribute to obesity or glucose intolerance. Whether the ER stress response is dependent on tissue type needs to be further explored. However, another study was performed in liver tissue where ER stress response failure was observed in obese and diabetic mice and patients. In a nonalcoholic fatty liver disease (NAFLD) or insulin resistance context, ER stress response failure is a causal factor contributing to the progression of obesity-associated diabetes or nonalcoholic steatohepatitis (NASH)^91^.

**Fig. 3:**
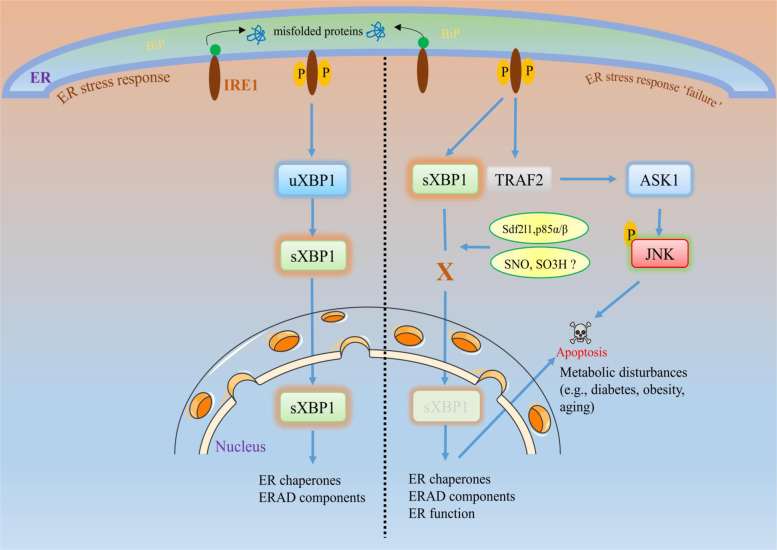
ER stress response failure and cellular fate. During acute or short-term ER stress, the cell follows its natural adaptive pathway (as explained in Fig. 1) to maintain cellular homeostasis. However, during prolonged ER stress or under certain conditions, such as aging or metabolic diseases (e.g., obesity or diabetes), the activated UPR sensors may not activate downstream signaling (here, we focus on IRE1 signaling). For example, failure of XBP1s to translocate to the nucleus to activate its target genes leads to decreased activation of XBP1s target genes, such as chaperones or ERAD. This diminished effect is called ER stress response failure, which may trigger apoptotic signaling rather than adaptive responses. Evidence of ER response failure in metabolic diseases suggests that the impaired interaction of XBP1s with the insulin receptor or the regulatory subunits of PI3K p85α and p85β blocks XBP1s translocation to the nucleus. Similarly, excessive reactive oxygen species and/or reactive nitrogen species-induced nitro-oxidative stress production induces sulfonation (SO3H) of IRE1 or SNO of IRE1α, which can decrease IRE1α ribonuclease activity, thereby inhibiting the production of XBP1s^88,108,160^. This impaired signaling may disrupt the ER chaperones, ERAD, or their functions, which may negatively affect cell survival and trigger apoptosis, leading to the subsequent disease progression.

Mainly, physiological disturbances in the ER cause ER stress response dysfunction in which various factors are involved. The exact cause of ER stress response failure is undetermined; however, the contributing factors are postulated to be involved under specific pathological conditions. For example, chronic stress increases ER dysfunction and leads to protein homeostasis diseases, such as aging-associated Alzheimer’s or Parkinson’s disease, metabolic diseases, amyotrophic lateral sclerosis, etc. Chaperones and foldases, such as GRP78 and PDI, play protective roles by reducing protein aggregation, increasing ER function, maintaining proteostasis, or reducing ER stress. Posttranslational modifications such as SNO, for instance, SNO modification of PDI, inactivate protein function and increase ER stress. The addition of a NO group to a cysteine residue within a protein is called a SNO modification, which has diverse functions, such as regulation of metabolism, cellular apoptosis, and transcription factors^92–94^. The excessive uncontrolled production of NO can induce cell death by several mechanisms. ROS-mediated oxidative stress generated from different sources, such as mitochondria or the ER, also leads to ER stress and cell death (discussed in the previous section). We can easily suggest that the nitro-oxidative stress induced by excessive ROS or reactive nitrogen species (RNS) may contribute to ER stress response failure-mediated cell death. However, it is surprising that the overproduction of NO-derived RNS (NO-RNS) can increase SNO-IRE1, which inhibits the endoribonuclease activity of IRE1 to inhibit XBP1 splicing. Since XBP1s acts as a transcription factor, the inhibition of XBP1s or its nuclear translocation may not sufficiently induce the expression of its target genes, such as ER chaperones or ERAD target genes (Fig. 3). ER chaperones or ERAD target genes are critical for maintaining ER homeostasis.

ROS play major roles in inducing the ER stress response and vice versa. Mitochondria were previously shown to be involved in inducing ROS production; however, accumulating evidence suggests that ROS originating from the ER have roles similar to those of mitochondria-derived ROS, disturbing ER protein folding, and generating ER stress^95^. ROS and RNS can stimulate oxidative stress and ER stress, potentially driving cellular apoptosis. Excessive ROS and RNS production from H_2_O_2_ or NO can cause nitro-oxidative stress and lead to ER stress-induced cell death^93,95,96^. Under this condition, severe tissue damage may be caused, which may lead to pathogenesis. Furthermore, the loss of antioxidants such as GSH, SOD, or catalase, or the disruption of disulfide bond formation in the ER can increase ROS generation and protein unfolding or misfolding in the ER, contributing to oxidative stress. Some of the contributors to the ER stress response pathway, such as the high activation of CHOP or ERO1α, can also induce ROS generation^96^. ROS produced by metabolic stress can trigger phospholipase C activation and ER calcium release, and can increase ER stress and mitochondrial dysfunction^97^. It is suggested that protein folding is highly redox reaction-dependent. Therefore, oxidative stress and ER stress are considered integral UPR components, where ROS generation can be either upstream or downstream targets of the UPR. We have already discussed the impact of ROS on the ER stress response; however, ER stress response failure is currently the focus of understanding the post-ER stress response and its interplay with redox signaling.

Mouse model investigations revealed high oxidative stress and dysregulated UPR responses (response failure) in the kidneys of aged mice after a high dose of tunicamycin injection. This failure was indicated by the loss of p-PERK and XBP1 splicing. The use of antioxidants prevented renal function failure by reducing oxidative stress and ER stress. Oxidative stress inhibition largely corrected the altered UPR in the aged kidneys and protected the old mice from a renal injury caused by a high dose of tunicamycin^98,99^. Similarly, in NAFLD, NASH, and pathological conditions of obesity and aging, the dysregulated expression of ER stress proteins and dysfunctional autophagy and apoptosis were observed. The expression of ER chaperones, such as GRP78, GRP94, or calnexin and foldases, such as PDI, ERp44, and ERp72 was reduced under NASH conditions. The transcription factors associated with ER stress, such as cleaved ATF6, spliced XBP1, and CHOP, were highly increased in NASH tissues. Dysregulated expression of BCL-2 family proteins was observed, where BCL-2 was highly upregulated, and BIM and MCL-1 were reduced significantly in NASH patients compared with control patients. These phenomena were correlated with the dysregulated expression of autophagy-related proteins, with high levels of Atg16L1 and LC3B found, and no significant differences were observed in the expression levels of Beclin 1, the Atg5–Atg12 conjugate, LC3A, or p62 in NASH tissues. These findings support the notion that ER stress response impairment/failure or autophagy dysregulation can induce severe pathologies, such as NASH, where the protein quality control system is highly compromised^100^. Several research groups also believe that UPR dysfunction is one of the major factors that contributes to the accumulation of disease-related proteins leading to pathogenesis, such as amyotrophic lateral sclerosis, Alzheimer’s disease, Parkinson’s disease, and Huntington’s disease^94,101,102^. Since the main sensors of the UPR are localized in the ER, the role of the ER is identified as the hub for regulating the UPR^102^.

The ER has a role in fundamental functions such as posttranslational modifications that affect proper folding, and assembly of individual subunits and oligomerization. The interaction with chaperone proteins plays a very important role at each cotranslational and posttranslational step. In addition, it plays a crucial role in catalyzing isomerization reactions, balancing the proteins in a folding-competent state and in degradation pathways^103,104^. Notably, several chaperones or foldases, such as GRP78 or PDI, reduce the accumulation of misfolded proteins. The best example is observed in neurodegenerative diseases, where the chaperones have a critical role in ameliorating the oxidative/nitrosative stress-induced misfolded proteins, representing an adaptive response of cells^93^. In Alzheimer’s or Parkinson’s disease, PDI is S-nitrosylated, which is induced by the excessive production of NO and NO-RNS. This modification leads to the inhibition of PDI enzymatic activity and the accumulation of polyubiquitinated proteins, thereby activating the UPR. Here, NO blocks the defensive effect of PDI, suggesting that the overproduction of NO-mediated SNO of PDI can be deleterious to the cell by promoting prolonged UPR activation and cell death^93,105,106^. In addition to PDI SNO, other ER stress pathway proteins also become S-nitrosylated, affecting the UPR in Parkinson’s disease. Excessive NO stimulates the SNO modification of ER stress sensors, such as IRE1α and PERK. When the ER stress sensors are S-nitrosylated, downstream elements are affected. For example, SNO-IRE1 inhibits ribonuclease activity and attenuates XBP1 splicing; however, it does not affect the phosphorylation or oligomerization of IRE1α, while the SNO of PERK activates its kinase activity, leading to the phosphorylation of its downstream target eIF2α. Researchers have demonstrated that the site-directed mutagenesis of IRE1α at cysteine 931 (Cys931), a predominant nitrosylation site in IRE1α, averts SNO and increases IRE1α ribonuclease activity^107,108^. SNO of IRE1 has also been observed in metabolic diseases, such as genetic and dietary models of obesity, where downstream XBP1 splicing activity is inhibited^108^. Under this condition, other canonical ER stress sensors, such as PERK and IRE1, are phosphorylated, and other ER stress-related events, such as JNK phosphorylation and UPR gene expression, are also increased. These data suggest that SNO of IRE1 inhibits the adaptive pathway of XBP1 but promotes the JNK pathway (upon its activation), which further damages hepatic cells by inducing apoptosis. Moreover, SNO of IRE1 alpha attenuated the downstream target ER chaperone genes of XBP1s, such as GRP78, PDI, and EDEM, and we suspect that the preventive effect of the ER chaperones may be abrogated during the NO-mediated SNO of IRE1α (ref. ^108^). Another report suggests that the inhibition of XBP1 increases oxidative stress, inflammation, and apoptosis in ob/ob mice, where JNK regulates the transition from adaptive to apoptotic UPR^109^.

A recent paper also demonstrated that Sdf2l1, an ER-resident molecule with a chaperoning function, was decreased in obese and diabetic mice^91^. These data were correlated with a decreased level of nuclear XBP1s, possibly because of the disruption of the ERAD pathway. An insufficient level of Sdf2l1 was also correlated with insulin resistance and steatohepatitis. The ER stress response pathway was also observed to be impaired in obesity and obesity-related diabetes, where the downstream target proteins of the main ER stress sensors were insufficiently activated. This impairment leads to ER stress response failure, which exacerbates ER stress. Furthermore, a decreased level of nuclear XBP1s is observed in diabetic patients and nephropathy murine models^110^. In this study, the authors observed a maladaptive ER stress response in the disease model, which indicated that simultaneously none of the UPR elements were activated; for instance, nuclear XBP1s was reduced and nuclear ATF6 and CHOP were increased. However, the mRNA expression of the UPR target genes that enhance the protein folding function of molecular chaperones (DNAJB9, DNAJC3, PDIA4, and Ero1b) and ERAD (Edem1) was increased in patients with diabetic nephropathy. These data suggest that the decreased nuclear translocation of XBP1s and the activation of its target genes are impaired during ER stress response failure or that the ER response does not depend on downstream XBP1 target genes. Nuclear XBP1 is impaired in diabetic mice and in cells treated with high levels of glucose, a finding confirmed by podocyte-specific deletion of XBP1, with its deficiency promoting ER stress in diabetic nephropathy. However, ATF6 overexpression in podocytes increased diabetic nephropathy through ATF6-dependent CHOP activation. These researchers explained that XBP1s lies downstream of insulin signaling and that the disturbance to insulin signaling or its sensitivity is caused by the impairment of the insulin receptor or the regulatory subunits of PI3K p85α and p85β. The interaction of XBP1s with p85α and p85β is critical to prevent diabetic nephropathy, and this disruption enhances pathogenesis. The pathogenesis of diabetic nephropathy and the disruption of XBP1s and P13K subunit interactions are also linked with obesity-associated insulin resistance. In an ob/ob mouse model, the interaction between p85 and XBP1s was lost in the liver, thereby reducing the nuclear translocation of XBP1s and inducing severe ER stress^111^. These data collectively suggest that nuclear XBP1 is critical for maintaining ER homeostasis, and its disruption can cause severe ER stress. The maladaptive ER stress response can limit the adaptive response and may follow ER stress-mediated cell death signaling. Interestingly, the same group of researchers found that bromodomain-containing protein 7 (BRD7), a tumor suppressor gene, is a component of UPR signaling and can regulate the nuclear translocation of XBP1. In addition, BRD7 interacts with the regulatory subunits of PI3K and enhances the nuclear translocation of p85α, p85β, and XBP1s. Furthermore, the in vivo data show that BRD7 expression was low in obese livers, and that its overexpression in the liver enhanced glucose homeostasis in diabetic and obese mice by restoring the nuclear translocation of XBP1s (ref. ^112^). Obesity needs to be controlled to maintain the health and lifespan because obesity accelerates the aging process^113^. In obesity, ER protein folding is impaired, and the UPR is induced, leading to hepatic steatosis, while overexpressing the ER chaperone (GRP78) prevents hepatic steatosis. Increased free fatty acids increase ROS and ER stress, and diminish SERCA activity. Several inflammatory modulators are also involved in obesity and its associated conditions. However, increased inducible nitric oxide synthase (iNOS) activity plays a major role in causing SNO of IRE1α, which further impairs ER function and prolongs ER stress^108^. Furthermore, iNOS induces ER stress-associated insulin resistance, where suppression or deletion of iNOS significantly enhances insulin sensitivity under HFD-fed conditions^108,114^. Similarly, an increase in misfolded proteins and loss of chaperones, or diminished proteasomal degradation are observed in aging and related pathologies, such as Parkinson’s or Alzheimer’s disease^93,115,116^. Subsequently, increased protein misfolding leads to cell death by enhancing apoptosis-inducing proteins^107,108^. For example, in a recent study, the loss of the ER chaperone GRP78 induced pulmonary fibrosis through increased ER stress, apoptosis, and senescence. Decreased GRP78 expression and increased ER stress-mediated apoptosis were revealed, as shown by increased CHOP and cleaved caspase-3 levels, suggesting that impaired ER stress or impaired UPR response reduces the function of old alveolar type II cells^117^.

The mechanisms described are promising for the study of ER stress response failure in metabolic diseases, where the downstream ER stress sensors fail to be fully activated. Further investigation of how ER stress response failure contributes to cell death during metabolic disturbances is needed. Considering the evidence, we can hypothesize that the expression of apoptotic proteins involved in ER stress is predominantly increased, whereas the expression of proteins involved in the adaptive response is diminished. In addition, ER stress response failure and its mechanism have been connected and applied to treating certain malignancies. Regarding potential therapies, protein disulfide isomerase inhibitors increased PERK dimerization and IRE1α oligomerization, decreasing the effect of inactive XBP1s on the accumulation of misfolded ER proteins^118^. The downstream signaling molecule of PERK-CHOP remained mostly intact. In other arms of the UPR, the upstream IRE1α was activated but not the downstream transcription factor XBP1s, which has a role in the adaptive ER stress response. The reduction in nuclear XBP1s was correlated with elevated poly (ADP-ribose) polymerase cleavage, suggesting the involvement of ER stress-mediated apoptosis^118^. These data provide strong evidence implicating ER stress response failure-mediated apoptosis signaling as a means to treat cancer phenotypes.

While ER stress has been linked with various diseases, such as diabetes, obesity, or aging, the specific regulatory mechanism of the UPR pathways that lead to pathogenesis still needs to be determined in further research. Whether the involvement of all UPR pathways is necessary to induce ER stress, or whether one specific pathway is enough to induce ER stress, the disease progression needs to be elucidated in future studies.
